# Immunopathology of and potential therapeutics for secondary hemophagocytic lymphohistiocytosis/macrophage activation syndrome: a translational perspective

**DOI:** 10.1038/s12276-024-01182-6

**Published:** 2024-03-06

**Authors:** Tram T. T. Nguyen, Yoon Tae Kim, Geunyeol Jeong, Mirim Jin

**Affiliations:** 1 Department of Health Sciences and Technology, GAIHST, Gachon University, Incheon, Republic of Korea; 2 Lee Gil Ya Cancer and Diabetes Institute, Gachon University, Incheon, Republic of Korea; 3 Department of Microbiology, College of Medicine, Gachon University, Incheon, Republic of Korea

**Keywords:** Autoimmunity, Infectious diseases, Tumour immunology

## Abstract

Secondary hemophagocytic lymphohistiocytosis/macrophage activation syndrome (sHLH/MAS) is a life-threatening immune disorder triggered by rheumatic disease, infections, malignancies, or medications. Characterized by the presence of hemophagocytic macrophages and a fulminant cytokine storm, sHLH/MAS leads to hyperferritinemia and multiorgan failure and rapidly progresses to death. The high mortality rate and the lack of specific treatments necessitate the development of a new drug. However, the complex and largely unknown immunopathologic mechanisms of sHLH/MAS, which involve dysfunction of various immune cells, diverse etiologies, and different clinical contexts make this effort challenging. This review introduces the terminology, diagnosis, and clinical features of sHLH/MAS. From a translational perspective, this review focuses on the immunopathological mechanisms linked to various etiologies, emphasizing potential drug targets, including key molecules and signaling pathways. We also discuss immunomodulatory biologics, existing drugs under clinical evaluation, and novel therapies in clinical trials. This systematic review aims to provide insights and highlight opportunities for the development of novel sHLH/MAS therapeutics.

## Battling the Cytokine Storm: A Deep Dive into sHLH/MAS Therapeutics

In the intricate realm of health issues, there’s a serious inflammation-related disorder known as secondary hemophagocytic lymphohistiocytosis, which can escalate to a potentially fatal situation called a cytokine storm. This condition involves the excessive activation of immune cells named macrophages and can lead to signs like constant fever, irregularities in blood cells, and organ enlargement. While sHLH can emerge from various infections and diseases, its precise causes and processes are not completely known, creating a void in our capability to diagnose and treat it effectively. This review is a thorough analysis of sHLH, concentrating on the immune system’s role in the disease and potential treatments. The significance of this research lies in its potential to enhance the diagnosis and management of sHLH, which could result in improved patient outcomes.

This summary was initially drafted using artificial intelligence, then revised and fact-checked by the author.

## Introduction

**Fig. 1:**
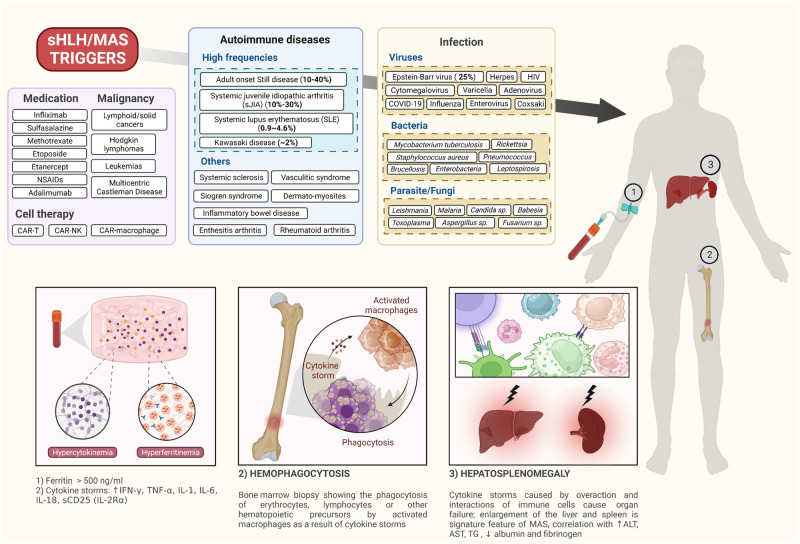
sHLH/MAS triggers and related incidences. (Upper panel) sHLH/MAS triggers are diverse and have different incidences. (Lower panel) Key clinical features of sHLH/MAS.

Secondary hemophagocytic lymphohistiocytosis (sHLH) is a hyperinflammatory condition characterized by the proliferation and accumulation of macrophage-like histiocytes, leading to cytokine storm, hemophagocytosis (phagocytosis of blood cell components via overactivation of macrophages)^1^, hyperferritinemia, persistent high-grade fever, cytopenia of at least two lineages, hepatosplenomegaly, coagulopathy, hypertriglyceridemia and multiorgan failure. In contrast, primary or familial HLH (fHLH) is associated with genetic defects, such as perforin gene mutation;^2^ however, sHLH has been identified as a complication triggered by a variety of infectious agents, including viruses, bacteria (such as *Rickettsia* spp.), fungi, and protozoa, as well as by hematologic malignancies^3^. Macrophage activation syndrome (MAS) is specifically associated with rheumatologic disorders such as systemic juvenile idiopathic arthritis (sJIA), systemic lupus erythematosus (SLE), Kawasaki disease, and adult-onset Still’s disease (AOSD)^4–7^. HLH-2004, a new diagnostic guideline, comprises a set of eight criteria: fever, splenomegaly, cytopenia, hypertriglyceridemia and/or hypofibrinogenemia, hemophagocytosis, low or absent natural killer (NK) cell activity, hyperferritinemia (a serum ferritin concentration greater than 500 ng/mL), and a high sIL-2R (sCD25) level^8^ (Fig. 1). Based on these variables, in 2014, the reactive hemophagocytic syndrome diagnostic score (HScore) was the first validated score that could be used for the detailed diagnosis of HLH^9^. Preliminary guidelines for MAS in patients with juvenile SLE (jSLE) aim to ensure timely diagnosis and accurate classification^10^. The 2016 EULAR criteria for sJIA/MAS distinguish patients with active sJIA from those with systemic infections^11^. In 2019, a simple MAS/sJIA score was developed to help physicians identify MAS in patients with active sJIA in a timely manner^12^ (Table 1).

**Table 1:** Diagnostic and classification criteria for sHLH/MAS.

	HLH		MAS		
	2004 HLH^8^	2014 HScore^9^	2009 MAS/jSLE^10^	2016 EULAR MAS/sJIA^11^	2019 MAS/sJIA (MS) score^12^
Fever (°C)	≥38.5	0 (<38.4), 33 (38.4–39.4), or 49 (>39.4)	≥38	Not specified	–
Hyperferritinemia (ng/mL)	≥500	0 (<2000), 35 (2000–6000), or 50 (>6000)	>500	>684	0.0001×value
Hypertriglyceridemia (mg/dL)	≥265	0 (<132.7), 44 (132.7-354), or 64 (>354)	>178	>156	–
Hypofibrinogenemia (g/L)	≤1.5	0 (>2.5) or 30 (≤2.5)	≤1.5	≤3.6	-0.004×value
Cytopenia	Platelets<100×10^9^/L, Hemoglobin<90 g/L, Neutrophils<1.0×10^9^/L	Platelets<110×10^9^/L, Hemoglobin<92 g/L, Leukocytes<5.0×10^9^/L	Platelets<150×10^9^/L, Hemoglobin<90 g/L, Leukocytes<4.0×10^9^/L	Platelets≤181×10^9^/L,	-0.003×platelets
Aspartate aminotransferase (AST) (U/L)	–	0 (<30) or 19 (≥30)	>40	>48	–
Lactate dehydrogenase (LDH) (U/L)	–	–	>567	–	0.001×value
NK cell activity	Low or absent	–		–	–
Soluble IL-2Rα (sCD25) (U/mL)	≥2400	–		–	–
Organomegaly	Splenomegaly	0 (N), 23 (hepato- or splenomegaly), or 38 (hepato- and splenomegaly)	Hepato- and splenomegaly (≥3 cm below the costal arch)	–	–
Hemorrhagic manifestations	–	–	Purpura, easy bruising, or mucosal bleeding	–	1.54×1(Y) or ×0(N)
Central nervous system involvement	–	–	Irritability, disorientation, lethargy, headache, seizures, or coma	–	2.44×1(Y) or ×0(N)
Active arthritis	–	–		–	-1.3×1(Y) or ×0(N)
Known immunosuppression	–	0 (N) or 18 (Y)		–	–
Hemophagocytosis	Bone marrow, spleen, or lymph nodes	0 (N) or 35 (Y) in bone marrow	Bone marrow aspirate	–	–
Diagnosis	Presence of genetic mutations or 5 of 8 criteria met	Sum of parameters ≥169	At least 1 clinical criterion + 2 laboratory criteria	Sum of parameters ≥-2.1	Fever in known or suspected sJIA + ferritin + 2 of 4 remaining criteria met

Without efficient treatment, the mortality of patients with sHLH/MAS is unacceptably high (20–53% and up to 70% in patients with specific types)^13^. The complexity of its immunopathology, which remains largely unexplained, poses significant challenges to new drug development. First, sHLH/MAS may be driven not only by innate immune responses or inflammatory diseases but also by a combination of other factors, including genetics, malignancy, cancer therapies, medications, and infections. The intricate interactions among various immune cells, cytokines, and chemokines linked to these underlying causes make identifying key biomarkers and therapeutic targets challenging^14,15^. Second, sHLH/MAS is heterogeneous, with cases spanning a spectrum from mild to severe, and can be triggered endogenously or exogenously in the presence or absence of infection (Fig. 1). In some patients, the etiology of and infectious agents contributing to sHLH/MAS are unknown. This variability leads to differing diagnostic criteria and patient recruitment in studies, resulting in outcomes that are difficult to interpret. Third, current animal models for sHLH/MAS are based on immunological stimulators such as infections, Toll-like receptor (TLR)-stimulating agents such as CpG, or persistent exposure to interleukin (IL)-6^16^. However, the immunopathologies induced by these triggers may differ from each other and from clinical etiologies^17,18^. Moreover, no animal model for sHLH associated with cancer has been established. These challenges significantly hinder the development of effective drugs for sHLH/MAS.

In this review, we cover the clinical characteristics, etiology, and immunopathological mechanisms of sHLH/MAS at both the cellular and molecular levels. Additionally, we examine the significance and limitations of current strategies for treating sHLH/MAS, providing insights from a translational perspective.

## Immunopathology of and potential therapeutics for sHLH/MAS

**Fig. 2:**
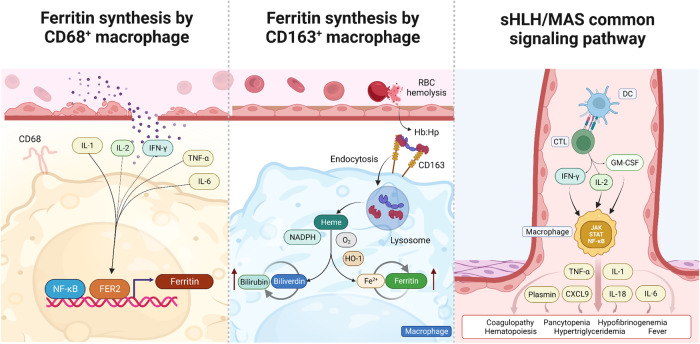
Etiology of sHLH/MAS. (Left) FER2, a transcription factor for the H chain, has a binding site located 4.8 kb upstream of the transcription start site of the ferritin gene and contains a binding site for the transcription factor NF-κB. Moreover, TNF-α, IL-2, IL-1, and IL-6 can increase the synthesis of ferritin. (Middle) Haptoglobin–hemoglobin (HP-Hb) complex uptake by macrophages via CD163 increases ferritin production. (Right) IFN-γ and IL-2 secreted by CTLs activate the JAK/STAT and NF-κB pathways in macrophages, which in turn produce numerous cytokines, leading to the manifestation of the clinical features of sHLH/MAS.

Although the specific immunopathologic mechanisms of sHLH/MAS are unknown, all patients with sHLH/MAS exhibit similar clinical features, predominantly hyperferritinemia and bone marrow abnormalities. Bone marrow abnormalities may be caused by aberrant histiocytosis, altering the bone marrow milieu and leading to a reduction in or cessation of hematopoietic stem cell generation. Consequently, pancytopenia occurs predominantly in patients with bone marrow failure, particularly in those with severe or advanced disease, and is characterized by reductions in the abundances of circulating red blood cells, white blood cells (WBCs), and platelets^19^. In the early phase, ferritin produced by CD68^+^/IL12^+^/CD163^low^ macrophages is detected in the bone marrow^20^. Simultaneously, the levels of cytokines and chemokines in the bloodstream increase. Tumor necrosis factor (TNF)-α, IL-2, IL-1β, and IL-6 upregulate ferritin synthesis via FER2, a transcription factor that binds a region located 4.8 kb upstream of the transcription start site of the ferritin gene and also contains a binding site for nuclear factor-κB (NF-κB) (Fig. 2, left panel)^20–22^. Lymphatic histiocytic infiltration is often found in the spleen, lymph nodes, and bone marrow^23^. This infiltration is followed by the uptake and phagocytosis of hemoglobin-haptoglobin complexes via CD163, a process that induces antioxidant responses that increase the ferritin content during disease progression, suggesting a change in macrophage polarization (Fig. 2, middle panel)^24^. Even if the etiologies may differ, all sHLH/MAS cases share common cytokine profiles and signaling pathways. The development of this disease appears to be mediated primarily by the release of interferon (IFN)-γ and IL-2 from activated cytotoxic T lymphocytes (CTLs) in response to stimulation by antigen-presenting cells (APCs). These cytokines subsequently trigger the activation of Janus kinases (JAKs), signal transducer and activator of transcription proteins (STATs), and NF-κB-dependent transcriptional signaling in macrophages to induce the production of TNF-α, IL-1, IL-18, IL-6, and chemokine (C-X-C motif) ligand 9 (CXCL9)^24^. Elevated levels of ILs may cause fever and hematopoiesis, IFN-γ and TNF-α might be involved in pancytopenia, and TNF-α may be related to hypertriglyceridemia via inhibition of lipoprotein lipase^25,26^. Hypofibrinogenemia, resulting from elevated blood levels of plasmin, develops due to increased production of plasminogen activators by activated macrophages, and liver dysfunction causes coagulopathy^27^ (Fig. 2, right panel).

The current treatment strategy for sHLH/MAS focuses primarily on relieving symptoms and addressing the causative factors. For instance, antibiotics and antivirals are used to treat cases with infectious etiologies. To combat hyperinflammation and overactivation of immune cells, high doses of steroids, nonsteroidal anti-inflammatory drugs, cyclosporine, and etoposide are used. sHLH/MAS treatment largely depends on glucocorticoids, with regimens often beginning with high doses of steroids (up to 30 mg/kg/d methylprednisolone, maximum dose: 1 g/day), followed by calcineurin inhibitors (cyclosporine A 2–7 mg/kg/day) and etoposide or other biologics for refractory cases^28^. The majority of the anti-inflammatory effects of glucocorticoids appear to result from transrepression, a key negative regulatory mechanism. In this process, a ligand-bound glucocorticoid receptor is recruited to chromatin through protein–protein interactions with DNA-bound transcription factors, particularly NF-κB and AP-1^29^. However, the treatment of some patients with sHLH/MAS, even those receiving high doses of corticosteroids, is challenging. The inevitable use of corticosteroids can lead to adverse effects such as obesity, osteoporosis, growth retardation, and, in some cases, infectious complications^28^. Studies in patients with refractory disease have explored the off-label use of biologics targeting inflammatory cytokines, including IL-1, IL-6, TNF-α, and IFN-γ; regulators of immune cells, including CTLA-4 in T cells and CD20 in B cells; and JAK1/2 inhibitors. Some of these treatments are undergoing clinical trials for sHLH/MAS.

In this paper, we provide an overview of the immunopathological mechanisms underlying sHLH/MAS and identify key molecules and signaling pathways as potential therapeutic targets.

### NK cell and CTL defects in patients with sHLH/MAS

**Fig. 3:**
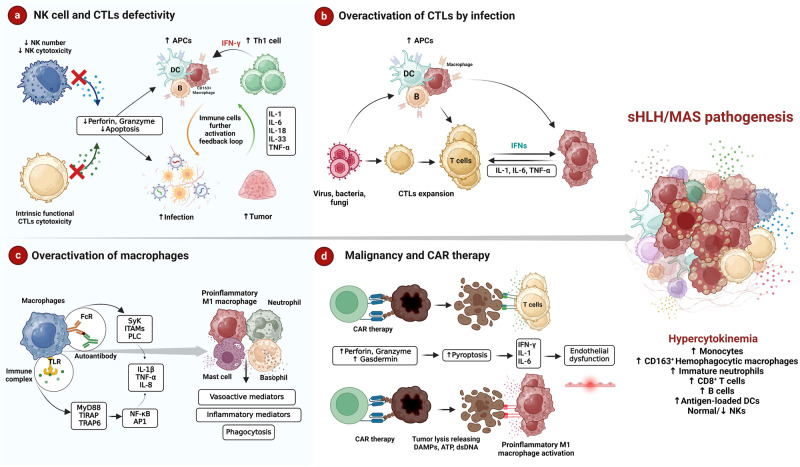
sHLH/MAS pathogenesis from a translational perspective. **a** NK cell and CTL defects and the consequent deficiency in perforin and granzyme secretion result in extended lymphocyte–APC interactions (with IFN-γ playing a key role) but an insufficient defense against infections and cancer, leading to the generation of a constitutive inflammatory loop via cytokines such as IL-1, IL-6, IL-18, IL-33, and TNF-α. **b** CTL proliferation as a result of innate immune activation by infection. Prolonging CTL–APC interactions results in the production and secretion of mediators (IL-1, IL-6, and TNF-α) that amplify coagulation and inflammation. Viral clearance by the host is ineffective despite the high levels of inflammatory cytokines because of the ability of viruses to escape host defenses. **c** An intense proinflammatory response mediated by macrophages may result from increased autoantibody and immune complex production, potentially leading to disease flares via the synergistic effects of TLRs and FcRs. TLRs activate MyD88, TIRAP, and TRAF6, ultimately activating transcription factors such as NF-κB and AP-1. FcRs activate Src family kinases that phosphorylate ITAMs and downstream signaling molecules such as PLC. IL-1, TNF-α, and IL-8 secretion leads to the recruitment of neutrophils for phagocytosis. TLRs may work in synergy with FcRIs on basophils and mast cells to induce the production of vasoactive and proinflammatory mediators. **d** To eliminate malignant cells, CAR-T cells release perforin, granzymes, and cytokines, leading to pyroptosis, which induces the release of DAMPs, ATP, endogenous dsDNA, RNA, histones, and nucleosomes. These proinflammatory substances cause sHLH/MAS by activating macrophages and CTLs.

The primary and most intensively studied mechanism of sHLH/MAS is the ineffectiveness of NK cells and CTLs in clearing abnormal cells, resulting in the uncontrolled proliferation of immune cells, such as T lymphocytes and APCs, that secrete large amounts of cytokines. NK cells and CTLs kill “stressed cells” through the following two mechanisms: (i) releasing lytic granules containing lysosomal hydrolases, perforin, and granzymes^30^ and (ii) inducing apoptosis in infected or malignant cells via TNF-related apoptosis-inducing ligand or Fas ligand^31^. Failure of either mechanism can lead to immunological proliferation and prolonged lymphocyte–APC interactions, but a weak defense against infection and cancer generates a persistent inflammatory loop that is usually observed in sJIA and AOSD-associated MAS^32^ (Fig. 3a). Kawasaki disease, characterized by unexplained swelling and inflammation of the internal vascular walls, can progress to MAS at any stage of the disease^33^. Research has shown that CTLs infiltrating the coronary artery lack the cytotoxic proteins perforin and granzyme. A defect in the expression of natural killer group 2 A (NKG2A), a protein that is constitutively expressed on all resting NK cells and CTLs and acts as a sensor to recognize “induced self” ligands for the elimination of aberrant cells, may be a contributing factor^6^.

Human NK cells are divided into two subgroups, each with distinct functional properties. CD56^dim^ NK cells are highly effective cytotoxic effector cells that release large amounts of perforin and relatively small amounts of cytokines. In contrast, CD56^bright^ NK cells have a high capacity to produce inflammatory cytokines, particularly IFN-γ, but are poor cytotoxic effectors^34^. For unknown reasons, low CD56^bright^ NK cell numbers and profound impairment of the cytotoxic activity of CD56^dim^ NK cells are observed in patients with sJIA and AOSD-associated MAS^35,36^. In patients with fHLH, mutations in genes that regulate granule-dependent cytotoxicity in CD56^dim^ NK cells (including *PRF1*, *UNC13D*, *RAB27a*, and *STX11*) can result in defective clearance of infected or tumor cells^37^.

Several drugs targeting this mechanism have been employed as sHLH/MAS therapeutics (Table 2). The first is etoposide, which directly and specifically acts by inducing apoptosis in CD4^+^ and CD8^+^ T cells, thereby reducing the IFN-γ concentration and macrophage overactivity. Interestingly, etoposide targets adaptive immune cells but does not affect naïve or memory T cells^38^. Another drug, alemtuzumab, which depletes mature T cells, has been demonstrated to result in positive clinical outcomes in patients with sHLH/MAS^39^. Cyclosporine, known for its ability to inhibit T cell activity, is used in the early stages of sHLH/MAS^40^. Furthermore, the anti-IFN-γ blocking antibody emapalumab has been approved by the FDA for the treatment of patients with fHLH with refractory, recurrent, or progressive disease or those intolerant to conventional HLH therapy; a phase II study on the use of emapalumab for sHLH/MAS treatment has recently been completed^17^ (Table 3). IFN-γ, a central and upstream factor in the pathogenesis of both fHLH and sHLH/MAS, promotes the release of cytokines such as TNF-α, IL-1, IL-6, CXCL9, and CXCL10 by activated macrophages, thereby exacerbating proinflammatory loops generated via defective NK cells and CTLs^41,42^. Like type II interferons, type I IFNs also signal through the JAK-STAT pathway and are most abundantly produced by NK cells, which influences their effector functions. However, the exact role of type I IFNs in sHLH/MAS is not yet clear. Since both NK cell cytotoxicity and IFN-γ production are influenced by type I IFNs, further research is needed to explore the role of type I IFNs in sHLH/MAS^41,42^.

**Table 2:** sHLH/MAS therapeutics reported in the literature.

Target	Drugs	Combined drugs	Condition	Trigger	Clinical and laboratory criteria	Results	Ref.
IL-1	Anakinra	Methylprednisolone, Ciclosporin, Prednisolone	sJIA	Methylprednisolone	↑Fever, ↑Splenomegaly, ↑Ferritin, ↓PLT, ↑AST, ↑Fibrinogen, ↑sCD25	Ferritin/PLT/AST/Fibrinogen normal range	^95^
		IVIG, Aspirin, Infliximab, Methylprednisolone, Prednisolone	KD, HLH	Methylprednisolone, Prednisolone	↑Ferritin, ↑WBC, ↑PLT, ↑AST, ↑ALT, ↑TG	Inflammatory marker recovery	^96^
		Methylprednisolone, Ciclosporin, Prednisolone, Dexamethasone	AOSD, HLH	Methylprednisolone	↑Fever, ↑Hepatomegaly, Cervical lymphadenopathy, ↑Neutrophilic leukocytosis, ↑Ferritin, ↑AST, ↑TG, ↑Hematopoietic cells	Ferritin/Inflammatory marker improvement	^97^
		Methylprednisolone, Corticosteroids, Tocilizumab	AOSD, HLH	Anakinra (100 mg/day), Methylprednisolone, Tocilizumab	↑Fever, ↑Hepatomegaly, ↑Splenomegaly, ↑Ferritin, ↓PLT, ↑AST, ↑ALT, ↓Leucocyte count, lymphopenia, ↑Hematopoietic cells	Ferritin/systemic inflammation and cytolysis improvement	^98^
		Methylprednisolone, Infliximab, Prednisolone, MTX, Dexamethasone	sJIA	Infliximab, Prednisolone, MTX	↑Fever, ↑Splenomegaly, ↑Ferritin, ↓PLT	Ferritin/WBC/PLT normal range	^99^
		Prednisone, Adalimumab	axSpA	Adalimumab	↑Fever, ↑Splenomegaly, Lymphopenia, ↓PLT, ↑AST, ↑ALT, ↑Ferritin	ESR/CRP/ferritin/fibrinogen/inflammatory marker improvement	^100^
	Canakinumab	Prednisone, Methylprednisolone, Anakinra, Cyclosporine A	sJIA	Prednisone, Methylprednisolone, Anakinra	↑Fever, ↑Ferritin, ↑PLT, ↑AST, ↑TG, ↑Fibrinogen	Ferritin decrease, AST normal range	^101^
		Methylprednisolone, Glucocorticoid, Cyclosporine A, Anakinra	sJIA	Prednisone, Thrombophlebitis	↑Fever, ↑Ferritin, ↓PLT, ↑AST, ↑ALT, ↑TG, ↓Fibrinogen	Improvements in all MAS biomarkers	^101^
IL-2	Daclizumab	Corticosteroids	HLH	-	↑Fever, ↑Ferritin, ↓WBC, ↓PLT, ↑AST, ↑ALT	Fever/Ferritin normal range	^102^
IL-6	Tocilizumab	Methotrexate, Prednisolone, Cyclosporine, Omalizumab, Anakinra	AOSD, HLH	EBV, Dexamethasone	↑Fever, ↑Ferritin, ↑Leukocytosis, ↓PLT, ↑AST, ↑TG, ↓ Fibrinogen	AOSD and idiopathic urticaria nonrecurrence	^103^
		Prednisolone, Ganciclovir, Sulfamethoxazole, Trimethoprim	AOSD	Prednisolone, Sulfamethoxazole, Trimethoprim, CMV	↑Fever, ↑Hepatomegaly, ↑Ferritin, ↓PLT, ↑AST, ↑ALT, ↑TG, ↓ Fibrinogen	Fever/skin rash improvement	^104^
IFN-γ	Emapalumab	Tocilizumab, Anakinra, Prednisolone, Corticosteroids, Alemtuzumab	sJIA	Parainfluenza 3 virus, Anakinra,	↑Fever, ↑Hepatomegaly, ↑Splenomegaly, ↑Ferritin, ↓WBC, ↓PLT, ↑AST, ↑ALT, ↑TG, ↑sCD25	Inflammatory marker/Ferritin/CXCL9 improvement	^105^
		Prednisolone, Methylprednisolone, Anakinra, Corticosteroids, Dexamethasone, Acyclovir, Fluconazole	AOSD	Prednisolone, Methylprednisolone, Anakinra	↑Fever, ↑Splenomegaly, ↑Ferritin, ↓PLT, ↑AST, ↑ALT, ↑TG, ↑Fibrinogen, ↑sCD25	Ferritin/PLT/Fibrinogen/TG improvement	^106^
		Etoposide, Dexamethasone, Rituximab, IVIG	HLH	EBV	↑Fever, ↑Hepatomegaly, ↑Splenomegaly, ↓WBC, ↓PLT, ↑Ferritin, ↓Fibrinogen, ↑sCD25,	Clinical symptom/ Ferritin/sCD25 level improvement	^17^
		Canakinumab, Sulfamethoxazole Methylprednisolone, Trimethoprim	sJIA	EBV	↑Fever, ↓PLT, Neutropenia, Lymphopenia, ↑Ferritin, ↑AST, ↓Fibrinogen,	Cytopenia/hypofibrinogenemia improvement	^61^
TNF-α	Etanercept	Ibuprofen, Solumedrol, Steroid, Prednisolone	sJIA	Ibuprofen	↑Fever, ↑Hepatomegaly, ↑Splenomegaly, ↑WBC, ↓PLT, ↑Ferritin, ↓PLT, ↑TG, ↓ Fibrinogen	Fever/Chest pain/Rash improvement	^107^
		Etoposide, Methylprednisolone, Prednisolone, Tacrolimus, MTX, Infliximab	AOSD	Prednisolone	↑Fever, ↑Ferritin, ↑WBC, ↓PLT, ↑AST, ↑TG, ↑Fibrinogen, ↑sCD25	Fever and polyarthralgia improvement	^108^
	Infliximab	IVIG, Acetylsalicylic acid, Methylprednisolone, Cyclosporine A	KD	IVIG, Acetylsalicylic acid	↑Fever, ↑Hepatomegaly, ↑Splenomegaly, ↑Ferritin, ↓PLT, ↑AST, ↑TG, ↓ Fibrinogen	Hepatomegaly/Splenomegaly/Clinical and Biochemical parameter improvement	^109^
		Dexamethasone, Etoposide, Infliximab, Daclizumab,	HLH	EBV, CMV, HHV6	↑Fever, ↑Hepatomegaly, ↑Splenomegaly, ↓WBC, ↓PLT, ↑Ferritin, ↑sCD25, ↑TG, ↑AST, ↑ALT,	Clinical symptom improvement	^110^
JAK 1/2	Ruxolitinib	Prednisolone, Tocilizumab Methylprednisolone, Anakinra,	AOSD	–	↑Fever, ↑Hepatomegaly, ↑Ferritin, ↓PLT, ↑AST, ↑TG, ↓ Fibrinogen	Blood counts/Ferritin/CD25 levels normal range	^111^
	Baricitinib	Glucocorticoid, Immunoglobulin, Cyclophosphamide, MTX, Prednisolone	Nodular panniculitis, HLH	Prednisolone	↑Fever, ↑Hepatomegaly, ↑Splenomegaly, ↑Ferritin, ↓PLT, ↑AST, ↑TG, ↓ Fibrinogen, ↑sCD25	Abnormal laboratory all improvement	^112^
CD20	Rituximab	Metronidazole, Etoposide, Cyclosporine, Dexamethasone	SLE	–	↑Fever, ↑Splenomegaly, ↓WBC, ↓PLT, ↑Ferritin, ↓PLT, ↑AST, ↑TG, ↓ Fibrinogen	Blood counts/Ferritin/TG all normal range	^113^
		Methylprednisolone, Prednisone, Tacrolimus, Cyclosporine A	DM	Methylprednisolone	↑Fever, ↑AST, ↑ALT, ↓WBC, ↓PLT, ↑Ferritin	Pancytopenia improvement	^114^
		Methylprednisolone, Immunoglobulin, rituximab, Prednisolone	XLP	EBV	↑Fever, ↑Hepatomegaly, ↑Splenomegaly ↑WBC, ↑AST	Clinical symptom improvement	^65^
CD28	Abatacept	Anakinra, MTX, Corticosteroids, Methylprednisolone, IVIG, Cyclosporine	sJIA	Anakinra, Corticosteroids	Development of chronic low-grade MAS with concurrent cutaneous vasculitis	Fever improvement, ESR normal range	^115^

**Table 3:** Biological therapies for sHLH/MAS in clinical trials.

Target	Drug	Type of Drug	Preclinical	Phase I	Phase II	Phase III	Identifier	Ref.
JAK 1/2	Ruxolitinib	JAK 1/2 inhibitors					NCT05137496	NA
	Methylprednisolone							
IL-1	Anakinra	IL-1R antagonists					NCT02780583	^116^
	Methylprednisolone							
IL-2	rhIL-2	Recombinant hIL-2 protein					NCT02569463	NA
IFN-γ	Emapalumab	Anti-IFNγ mAb					NCT03311854	NA
IL-1	Anakinra	IL-1R antagonist					NCT04339712	NA
IL-6	Tocilizumab	Anti-IL-6 mAb						
IL-18	Tadekinig alfa	IL-18BP					NCT03512314	NA
IL-2R	Rituximab	Anti-CD20 mAb					NCT05384743	NA
IL-1	Anakinra	IL-1R antagonist					NCT03332225	^117^
IFNγ-1β	Imukin	Recombinant hIFNγ-1b protein						

IL-18, a member of the IL-1 cytokine superfamily, is present in inflammasomes as a precursor protein and, similar to IL-1 and IL-33, is activated upon cleavage by caspase 1^43^. Extremely high IL-18 levels in the plasma and synovial fluid of patients with systemic-onset sJIA correlate positively with disease activity, and decreased NK cell numbers correlate positively with the IL-18 level in patients with MAS^44,45^. IL-18 is the most effective cytokine for regulating NK cells, and exposure to large amounts of IL-18 induces NK cell death^46^. The presence of free IL-18 concentrations of up to 100,000 ng/mL assists in distinguishing MAS from other autoinflammatory diseases^47^. Therefore, the use of IL-18BP, a natural antagonist of IL-18, or an anti-IL-18 neutralizing antibody should be investigated in sJIA-associated MAS, as this type of MAS is characterized by the positive correlation of the IL-18 level with NK cell dysfunction, which is not observed in fHLH^48^. The administration of rIL-18BP in anakinra- and infliximab-nonresponsive patients yielded positive results after timely monitoring of the IL-18 concentration^49^ (Table 3). Additionally, daclizumab, an anti-IL-2Rα-chain (CD25) blocking antibody that has shown success in treating multiple sclerosis (MS), exerts its effect via circulatory NK cells and indirectly regulates autologous CD4^+^ and CD8^+^ T-cell numbers via granzyme activity^50,51^. Daclizumab has resulted in positive responses in adult patients with fHLH; hence, its clinical investigation in sHLH/MAS is warranted (Table 2).

Finally, blockade of killer cell immunoglobulin-like receptor (KIR) and NKG2A, two inhibitory receptors that shape the cytotoxic function of NK cells, may be potential targets for treatment^52,53^. An antibody against NKG2A (monalizumab) was shown to restore the cytotoxic potential of NK cells isolated from patients with chronic lymphocytic leukemia^54^. In a phase I clinical trial, an antibody against KIR increased NK cell cytotoxicity to some degree^54^. Unfortunately, clinical trials assessing the safety and effectiveness of these antibodies for treating sHLH/MAS are currently lacking.

### Overactivation of CTLs by infection in patients with sHLH/MAS

Infection is a common trigger for sHLH/MAS. Epstein–Barr virus (EBV), herpesviruses, Leishmania, influenza virus, immunosuppression caused by antiretroviral therapy for HIV treatment and, more recently, SARS-CoV-2 (which causes COVID-19) are the most common causes of infection^55,56^. The most vulnerable patients with H1N1 influenza, H5N1 influenza, or COVID-19 were found to succumb to sHLH/MAS^57^. The breakdown of tolerance to self-antigens, driven by IFNs stimulated by exogenous antigens presented by APCs to CTLs, is considered a critical mechanism in this type of sHLH/MAS and leads to rapid and massive multiorgan dysfunction^1^. Infection activates innate immunity and in turn causes CTL proliferation, and high expression levels of CD25 and exhaustion markers (PD1 and CD95) on CTLs indicates that these cells constitute a homogeneous and highly active subset of cells^58^. In patients with sHLH, the number of circulating activated CD38^high^/HLADR^+^ CTLs increases, a parameter that can be used to distinguish patients with early sepsis^58^. Furthermore, laboratory indicators, including ferritin, hemoglobin, and lactate dehydrogenase levels, are related to the proportion of CD38^high^/HLADR^+^ CTLs^58^. Prolonging the CTL–APC interaction results in the synthesis and release of mediators that promote inflammation and coagulation. In most cases, type II IFNs drive these effects; however, type I IFNs are crucial in COVID-19^59,60^. However, despite the high levels of inflammatory cytokines, the host fails to eliminate viruses because of their capacity to evade host immune responses. This phenomenon leads to immune exhaustion with decreased IFN levels at the later stage of infection and further activates the complement system of innate immunity by an unknown mechanism, worsening the situation (Fig. 3b).

EBV, the most common pathogen triggering sHLH/MAS, typically infects B cells via CD21 and replicates within these cells; it can also infect NK and T cells. Acute EBV infection causes fulminant life-threatening sHLH/MAS, and persistent infection can result in transformation into malignancy^61,62^. Owing to the proliferation of EBV-activated CTLs, acute EBV-sHLH/MAS evokes fever, hepatosplenomegaly, lymphadenopathy, atypical lymphocytosis, and pharyngitis, a constellation of symptoms clinically defined as infectious mononucleosis^63^. Intravenous immune globulin (IVIG) has been widely used to treat infection-associated MAS^64^. In addition, rituximab (anti-CD20) is a B-cell-directed therapeutic agent that acts by reducing the number of circulating B cells for six months or longer and may prevent the lymphoma transformation observed in treated patients, explaining its success in EBV-associated sHLH/MAS^65^ (Table 2). After infection by the influenza virus or human cytomegalovirus, dendritic cells further migrate to the thymus and destroy thymic cells^66^, allowing the invading virus to govern antigen presentation, a phenomenon involved in HLH pathogenesis^67^. Regarding SARS-CoV-2, a postmortem histological analysis of bone marrow from patients showed an association of infection with a diagnosis of sHLH/MAS, and elevated circulating ferritin correlated with poor clinical outcomes in a retrospective study^68,69^. This may be due to the overresponsiveness of CD4^+^ and CD8^+^ T cells, which secrete macrophage-activating cytokines such as IFN-γ and granulocyte-macrophage colony-stimulating factor (GM-CSF). GM-CSF serves as a chemoattractant for the migration of monocytes and neutrophils into tissues. Moreover, GM-CSF induces proinflammatory macrophage polarization, which results in the production of numerous cytokines and chemokines^70^. At this early stage, IL-1RA, IL-10, and chemokine (C-C motif) ligand 5 (CCL5) are expressed in mild cases but not in severe cases. GM-CSF and TNF-α levels did not differ between patients with mild and severe disease. However, the IL-6 and IFN-γ levels increase during the late stage of severe illness^71^. In addition to activating T cells, SARS-CoV-2 can directly target macrophages. SARS-CoV-2 may inhibit type I IFN signaling to hijack the host defense via the viral ORF6, ORF8, and N proteins, leading to compromised immune responses with decreases in T-cell and NK cell populations in the late stage^72^.

In infection-associated sHLH/MAS, the dual role of cytokines in both hypercytokinemia and pathogen clearance needs to be considered when treating patients. For example, although IFNs play critical roles in SARS-CoV-2-induced hyperinflammation, the administration of recombinant type I IFN in patients with SARS-CoV-2 was reported to decrease viral replication and protein synthesis. Furthermore, triple antiviral therapy with lopinavir–ritonavir, ribavirin, and IFN-1β is reportedly more effective than lopinavir alone in patients with mild or moderate disease, indicating that combating virus overload is more beneficial in these patient populations than in those with severe disease^73^. Similarly, a clinical study assessing the potential effects of inhaled and intravenous GM-CSF is currently underway^74^. The potential benefits of anti-GM-CSF administration in patients with COVID-19 have also been reported, as GM-CSF is a key player in macrophage regulation and generates an autocrine/paracrine feedback loop driving cytokine storm^75^. Based on these observations, infection-triggered sHLH/MAS should be monitored carefully, considering the dual role of cytokines and chemokines at specific stages, during which timing may be the critical factor in determining whether pathogen clearance or inflammation control is most beneficial.

Some patients with severe sepsis develop a syndrome with symptoms similar to those of MAS called macrophage activation-like syndrome (MALS), and a classification system for the early diagnosis of MALS has been developed^76^. Specifically, the diagnosis of MALS applies to patients defined by the Third International Consensus Definitions for Sepsis and Septic Shock (Sepsis-3)^77^ and those with an HScore greater than 151 who also present with both hepatobiliary dysfunction and disseminated intravascular coagulation. However, these patients do not have hemophagocytosis, which is one of the criteria considered for the diagnosis of MAS^76^.

### Activation of macrophages by immune complexes and autoantibodies

Macrophages use two types of immunological receptors: TLRs, which initiate the innate immune response, and Fc receptors (FcRs), which serve as sensors for the adaptive immune response. The main signaling molecules of most TLRs are MyD88, TIRAP, TRAF6, and transcription factors such as NF-κB and activator protein 1 (AP-1)^78^. FcR activation leads to the phosphorylation of immunoreceptor tyrosine-based activation motifs (ITAMs) by Src family kinases, which, in turn, activates phospholipase C (PLC), intracellular calcium flux, and NADPH production. The combined activation of TLRs and FcRs controls cytokine synthesis via these pathways, thereby shaping inflammatory immune responses^78^. At the early stage, damage-associated molecular patterns (DAMPs) bind to TLRs; however, low levels of circulating IgG molecules may result in strong activation of MyD88-dependent TLR signaling but weak activation of ITAM-dependent Syk signaling, leading to only partial activation of macrophages. In contrast, macrophage interactions with the same DAMPs opsonized with several IgG molecules promote TLR–FcR synergy via both pathways to drive stronger proinflammatory polarization, which is essential for preferential upregulation of TNF-α, IL-8, and IL-1β. This phenomenon, in turn, leads to the recruitment of neutrophils for phagocytosis and promotes the expression of TLRs that act with FcRIs on basophils and mast cells to induce the production of vasoactive mediators^78,79^ (Fig. 3c).

Autoimmune disorders, such as SLE, frequently cooccur with sHLH/MAS and may be caused by the autoantibodies and immune complexes that trigger inflammatory responses in macrophages^80,81^. Additionally, through changes occurring during drug therapy, including drug-induced lupus, and the use of biologics, such as anti-TNF-α and anti-IL-6 antibodies, sHLH/MAS may be initiated through increases in the levels of autoantibodies that induce an intense proinflammatory response in macrophages, leading to disease flares^80,81^. Although TLRs and FcRs may be simultaneously activated in autoimmune diseases and sHLH/MAS, the effects of their joint activation have received little attention. In inflammatory autoimmune disorders, chronic activation caused by this crosstalk may be detrimental^79^. Recently, it has been shown that coligation of TLR4 and FcR with autoantibodies can change lipid rafts and alter the process of TLR4 dimerization and clustering in these regions^78^. By remodeling macrophage lipid rafts with either methyl-cyclodextrin or filipin, the IL-6 signaling response to TLR2–FcR coengagement is significantly altered. Analysis of the TLR2/4-induced phosphoproteome in macrophages revealed several sites phosphorylated via TLR2/4 ligation in proteins in the FcR-mediated phagocytosis pathway, demonstrating the ability of TLR2/4 signaling to increase FcR-mediated phagocytosis^82^. Endogenous/exogenous immune complexes and autoantibodies that activate FcRs in conjunction with TLRs seem to push macrophage polarization to the extreme end of the spectrum.

High-dose steroid treatment is the cornerstone treatment for this type of sHLH/MAS, and in cases with steroid resistance, an anti-FcRIII antibody or IVIG can be further used. The mechanism of action of IVIG has been previously demonstrated^83^. IVIG can induce FcRIIB expression in macrophages, preventing FcRIII-mediated clearance of IgG-opsonized platelets by cytotoxic antibodies and protecting against autoantibody-induced inflammation. Additionally, blocking FcRIIA on monocytes/macrophages strongly suppresses the synergistic secretion of TNF-α mediated by TLR–FcR crosstalk. An engineered human IgG1 backbone has been used to create a humanized version (Hu 15C1) of a mouse antibody that engages FcRI and FcRIIA, boosting its inhibitory potency toward inflammatory cells to block TLR4 activation in lipid rafts, suggesting possible modulation of TLR–FcR crosstalk^84^. Owing to the rarity of this form of sHLH/MAS, there are few reports examining the relationship between specific candidate cytokines and TLR–FcR coengagement events.

### Association of sHLH/MAS with malignancy and chimeric antigen receptor (CAR) therapy

Lymphoid cancers (most commonly affecting NK, T, and B cells) and CAR therapies (CAR-T, CAR-NK, and CAR-macrophage) can lead to a high incidence of sHLH in affected patients (up to 46%)^85^. Accumulating evidence suggests that anti-CD19 CAR-T-cell infusion causes sHLH/MAS as part of its toxicity spectrum. Moreover, HLH-like expression patterns have been observed after anti-CD22 CAR-T-cell therapy^86,87^. CAR cells recognize antigens on tumor cells via CD40-CD40L and release massive amounts of perforin, granzymes, and cytokines to cause pyroptosis in cancerous cells. Pyroptosis is characterized by cellular swelling, lysis, and release of proinflammatory cellular contents via gasdermin B and E, which are cleaved by granzymes A and B, respectively^88,89^ (Fig. 3d). Compared to apoptosis induced by CTLs, which is characterized by the release of lower amounts of perforin and granzymes, wherein perforin pores can self-repair to prevent cellular rupture, CAR cell therapy induces a surge in perforin and gasdermin secretion that causes damage exceeding the repair capacity, leading to the release of DAMPs, ATP, endogenous dsDNA, RNA, histones, and nucleosomes. These proinflammatory products activate both CTLs and macrophages, causing sHLH/MAS^88^. Furthermore, cleaved gasdermin activates a complex of protease cofactors in the coagulation cascade, which is initiated by the externalization of phosphatidylserine on the cell membrane when nanopores are formed^88,89^. Even though this type of sHLH/MAS is linked to amplified activity of common proinflammatory signaling pathways, functional NK cell deficiency has not been observed in these patients^90^. The extremely severe hyperinflammatory status in CAR therapy-associated sHLH correlates with ferritin concentrations in excess of 10,000 ng/mL, as observed in patients treated with CD19 CAR-T cells, and in excess of 100,000 ng/mL in patients treated with CD22 CAR-T and MCMA-targeted CAR-T cells^90^.

Recent treatment recommendations for sHLH/MAS include corticosteroids with or without biologics; anakinra is often the first-line treatment because of its acceptable side effect profile, and ruxolitinib and emapalumab are the next-line agents^91^. Given the role of IL-6, tocilizumab (TCZ) has been utilized, although its results in patients with sHLH/MAS triggered by CAR therapy have been inconsistent^92^. TCZ was effective in a few case reports of reactive sHLH/MAS, but a larger study comparing the effectiveness and safety of IL-6R inhibition to conventional therapies found that a significant proportion of patients in the TCZ group experienced disease progression and had lower survival rates than did those in the control group^91^. In clinical practice, patients are often treated with a combination of anakinra and TCZ^93^. Notably, IL-1 release from inflammatory monocytes precedes IL-6 release by several hours, and IL-1 can induce the secretion of IL-6 as well as that of soluble IL-6R (sIL-6R), suggesting that cytokine release syndrome (CRS) may be initiated primarily by IL-1 release^94^. However, further randomized studies are needed to fully understand the efficacy and safety of TCZ for use in treating sHLH/MAS.

## Conclusion and perspectives

Given the multiple triggers and clinical scenarios associated with sHLH/MAS, establishing a biomarker-based diagnostic approach and pinpointing targeted therapies remain challenging. Artificial intelligence-based algorithms integrating etiology, cytokine profiles, and biochemical and clinical parameters could offer precise diagnostic solutions, facilitate accurate patient identification, and be used to monitor disease progression. However, real-time informatics is not currently feasible.

Since mortality in patients with sHLH/MAS is often attributed to an overwhelming cytokine storm, most biologics that inhibit proinflammatory cytokine production and release have undergone clinical trials. In certain scenarios, combining biologics with immunosuppressive drugs enhances the effectiveness of immunosuppressive drugs. However, as each cytokine’s activity and contribution vary depending on the disease stage and the patient’s condition, a precision medicine strategy is essential. Cytokine monotherapy might be ineffective, as it fails to fully inhibit the cytokine cascade, potentially leading to dyshomeostasis of cytokine networks.

Therefore, targeting the upstream triggers of cytokine storm in patients with sHLH/MAS could represent a new pharmacological approach, potentially reducing inflammation severity without disrupting the cytokine network. The identification of diagnostic strategies and development of new treatments for sHLH/MAS are continuously evolving. Undoubtedly, further research into the underlying pathologic mechanisms and systematic translational research, including both retrospective and controlled studies of sHLH/MAS under specific conditions, are crucial. These efforts could provide future perspectives and solutions for precision medicine.

## References

[1] McGonagle D, Ramanan AV, Bridgewood C (2021). Immune cartography of macrophage activation syndrome in the COVID-19 era. Nat. Rev. Rheumatol..

[2] Goransdotter Ericson K (2001). Spectrum of perforin gene mutations in familial hemophagocytic lymphohistiocytosis. Am. J. Hum. Genet.

[3] Ramos-Casals M, Brito-Zeron P, Lopez-Guillermo A, Khamashta MA, Bosch X (2014). Adult haemophagocytic syndrome. Lancet.

[4] Grom AA, Passo M (1996). Macrophage activation syndrome in systemic juvenile rheumatoid arthritis. J. Pediatr..

[5] Borgia RE, Gerstein M, Levy DM, Silverman ED, Hiraki LT (2018). Features, Treatment, and Outcomes of Macrophage Activation Syndrome in Childhood-Onset Systemic Lupus Erythematosus. Arthritis Rheumatol..

[6] Garcia-Pavon S, Yamazaki-Nakashimada MA, Baez M, Borjas-Aguilar KL, Murata C (2017). Kawasaki Disease Complicated With Macrophage Activation Syndrome: A Systematic Review. J. Pediatr. Hematol. Oncol..

[7] Arlet JB (2006). Reactive haemophagocytic syndrome in adult-onset Still’s disease: a report of six patients and a review of the literature. Ann. Rheum. Dis..

[8] Henter JI (2007). HLH-2004: Diagnostic and therapeutic guidelines for hemophagocytic lymphohistiocytosis. Pediatr. Blood Cancer.

[9] Fardet L (2014). Development and validation of the HScore, a score for the diagnosis of reactive hemophagocytic syndrome. Arthritis Rheumatol..

[10] Parodi A (2009). Macrophage activation syndrome in juvenile systemic lupus erythematosus: a multinational multicenter study of thirty-eight patients. Arthritis Rheum..

[11] Ravelli A (2016). 2016 Classification Criteria for Macrophage Activation Syndrome Complicating Systemic Juvenile Idiopathic Arthritis: A European League Against Rheumatism/American College of Rheumatology/Paediatric Rheumatology International Trials Organisation Collaborative Initiative. Arthritis Rheumatol..

[12] Minoia F (2019). Development and initial validation of the MS score for diagnosis of macrophage activation syndrome in systemic juvenile idiopathic arthritis. Ann. Rheum. Dis..

[13] Rouphael NG (2007). Infections associated with haemophagocytic syndrome. Lancet Infect. Dis..

[14] Khan HH, Ansar I, Kontos N, Kumar S, Lyons H (2020). Report of a Fatal Case of Hemophagocytic Lymphohistiocytosis Syndrome and a Review of the Literature. Cureus.

[15] Andersson U (2021). Hyperinflammation: On the pathogenesis and treatment of macrophage activation syndrome. Acta Paediatr..

[16] Behrens EM (2011). Repeated TLR9 stimulation results in macrophage activation syndrome-like disease in mice. J. Clin. Invest.

[17] Lounder DT, Bin Q, de Min C, Jordan MB (2019). Treatment of refractory hemophagocytic lymphohistiocytosis with emapalumab despite severe concurrent infections. Blood Adv..

[18] Brisse E, Wouters CH, Matthys P (2015). Hemophagocytic lymphohistiocytosis (HLH): A heterogeneous spectrum of cytokine-driven immune disorders. Cytokine Growth Factor Rev..

[19] Keohane, E. M. et al. *Rodak’s hematology : clinical principles and applications*, Sixth edition. edn. Elsevier: St. Louis, Missouri, 2020.

[20] Ruscitti P (2016). H-ferritin and CD68(+) /H-ferritin(+) monocytes/macrophages are increased in the skin of adult-onset Still’s disease patients and correlate with the multi-visceral involvement of the disease. Clin. Exp. Immunol..

[21] Pham CG (2004). Ferritin heavy chain upregulation by NF-kappaB inhibits TNFalpha-induced apoptosis by suppressing reactive oxygen species. Cell.

[22] Ruscitti P (2015). Increased level of H-ferritin and its imbalance with L-ferritin, in bone marrow and liver of patients with adult onset Still’s disease, developing macrophage activation syndrome, correlate with the severity of the disease. Autoimmun. Rev..

[23] Billiau AD, Roskams T (2005). Van Damme-Lombaerts, R., Matthys, P. & Wouters, C. Macrophage activation syndrome: characteristic findings on liver biopsy illustrating the key role of activated, IFN-gamma-producing lymphocytes and IL-6- and TNF-alpha-producing macrophages. Blood.

[24] Crayne CB, Albeituni S, Nichols KE, Cron RQ (2019). The Immunology of Macrophage Activation Syndrome. Front Immunol..

[25] Grunfeld C, Gulli R, Moser AH, Gavin LA, Feingold KR (1989). Effect of tumor necrosis factor administration in vivo on lipoprotein lipase activity in various tissues of the rat. J. Lipid Res.

[26] Lin FC (2014). IFN-gamma causes aplastic anemia by altering hematopoietic stem/progenitor cell composition and disrupting lineage differentiation. Blood.

[27] Yin G (2020). The prognostic role of plasma fibrinogen in adult secondary hemophagocytic lymphohistiocytosis. Orphanet J. Rare Dis..

[28] Cassidy, J. T., Petty, R. E., Laxer, R. M. & Lindsley, C. B. *Textbook of pediatric rheumatology E-Book*. Elsevier Health Sciences, 2010.

[29] Ramamoorthy S, Cidlowski JA (2016). Corticosteroids: Mechanisms of Action in Health and Disease. Rheum. Dis. Clin. North Am..

[30] Ambrose AR, Hazime KS, Worboys JD, Niembro-Vivanco O, Davis DM (2020). Synaptic secretion from human natural killer cells is diverse and includes supramolecular attack particles. Proc. Natl Acad. Sci. USA.

[31] Zamai L (1998). Natural killer (NK) cell-mediated cytotoxicity: differential use of TRAIL and Fas ligand by immature and mature primary human NK cells. J. Exp. Med.

[32] Carter SJ, Tattersall RS, Ramanan AV (2019). Macrophage activation syndrome in adults: recent advances in pathophysiology, diagnosis and treatment. Rheumatol. (Oxf.).

[33] Noval Rivas M, Arditi M (2020). Kawasaki disease: pathophysiology and insights from mouse models. Nat. Rev. Rheumatol..

[34] Villanueva J (2005). Natural killer cell dysfunction is a distinguishing feature of systemic onset juvenile rheumatoid arthritis and macrophage activation syndrome. Arthritis Res Ther..

[35] Zhou J, Tang X, Ding Y, An Y, Zhao X (2013). Natural killer cell activity and frequency of killer cell immunoglobulin-like receptors in children with different forms of juvenile idiopathic arthritis. Pediatr. Allergy Immunol..

[36] Shimojima Y (2019). Characteristics of Circulating Natural Killer Cells and Their Interferon-gamma Production in Active Adult-onset Still Disease. J. Rheumatol..

[37] Vastert SJ (2010). Mutations in the perforin gene can be linked to macrophage activation syndrome in patients with systemic onset juvenile idiopathic arthritis. Rheumatol. (Oxf.).

[38] Johnson TS (2014). Etoposide selectively ablates activated T cells to control the immunoregulatory disorder hemophagocytic lymphohistiocytosis. J. Immunol..

[39] Havari E (2014). Impact of alemtuzumab treatment on the survival and function of human regulatory T cells in vitro. Immunology.

[40] Jenkins MK, Schwartz RH, Pardoll DM (1988). Effects of cyclosporine A on T cell development and clonal deletion. Science.

[41] Gothe F (2022). Aberrant inflammatory responses to type I interferon in STAT2 or IRF9 deficiency. J. Allergy Clin. Immunol..

[42] Platanias LC (2005). Mechanisms of type-I- and type-II-interferon-mediated signalling. Nat. Rev. Immunol..

[43] Arend WP, Palmer G, Gabay C (2008). IL-1, IL-18, and IL-33 families of cytokines. Immunol. Rev..

[44] de Jager W (2007). Blood and synovial fluid cytokine signatures in patients with juvenile idiopathic arthritis: a cross-sectional study. Ann. Rheum. Dis..

[45] Mazodier K (2005). Severe imbalance of IL-18/IL-18BP in patients with secondary hemophagocytic syndrome. Blood.

[46] Takakura M, Shimizu M, Yakoyama T, Mizuta M, Yachie A (2018). Transient natural killer cell dysfunction associated with interleukin-18 overproduction in systemic juvenile idiopathic arthritis. Pediatr. Int.

[47] Weiss ES (2018). Interleukin-18 diagnostically distinguishes and pathogenically promotes human and murine macrophage activation syndrome. Blood.

[48] Yasin S (2020). IL-18 as a biomarker linking systemic juvenile idiopathic arthritis and macrophage activation syndrome. Rheumatol. (Oxf.).

[49] Canna SW (2017). Life-threatening NLRC4-associated hyperinflammation successfully treated with IL-18 inhibition. J. Allergy Clin. Immunol..

[50] Jiang W, Chai NR, Maric D, Bielekova B (2011). Unexpected role for granzyme K in CD56bright NK cell-mediated immunoregulation of multiple sclerosis. J. Immunol..

[51] Bielekova B (2006). Regulatory CD56(bright) natural killer cells mediate immunomodulatory effects of IL-2Ralpha-targeted therapy (daclizumab) in multiple sclerosis. Proc. Natl Acad. Sci. USA.

[52] Lanier LL (2000). The origin and functions of natural killer cells. Clin. Immunol..

[53] Robbins SH (2007). Natural killer cells promote early CD8 T cell responses against cytomegalovirus. PLoS Pathog..

[54] Benson DM (2015). A Phase I Trial of the Anti-KIR Antibody IPH2101 and Lenalidomide in Patients with Relapsed/Refractory Multiple Myeloma. Clin. Cancer Res.

[55] Weitzman S (2011). Approach to hemophagocytic syndromes. Hematol. Am. Soc. Hematol. Educ. Program.

[56] Bracaglia C, Prencipe G, De Benedetti F (2017). Macrophage Activation Syndrome: different mechanisms leading to a one clinical syndrome. Pediatr. Rheumatol. Online J..

[57] Shrestha B, Omran A, Rong P, Wang W (2015). Report of a Fatal Pediatric Case of Hemophagocytic Lymphohistiocytosis Associated with Pandemic Influenza A (H1N1) Infection in 2009. Pediatr. Neonatol..

[58] De Matteis A (2022). Expansion of CD4dimCD8+ T cells characterizes macrophage activation syndrome and other secondary HLH. Blood.

[59] Lee JS, Shin EC (2020). The type I interferon response in COVID-19: implications for treatment. Nat. Rev. Immunol..

[60] Otsuka R, Seino KI (2020). Macrophage activation syndrome and COVID-19. Inflamm. Regen..

[61] Macaraeg M, Schulert GS (2023). Complications of complications: diagnosis and treatment of recurrent macrophage activation syndrome in a patient with well-controlled systemic juvenile idiopathic arthritis. RMD Open.

[62] Zehr B, Brannock K, Wyma R, Kahwash SB (2023). Differentiating fulminant EBV infection complicated by HLH from Lymphoma: report of a case and a brief literature review. Diagn. Pathol..

[63] Miyawaki T (1991). Expression of CD45R0 (UCHL1) by CD4+ and CD8+ T cells as a sign of in vivo activation in infectious mononucleosis. Clin. Exp. Immunol..

[64] Yaqinuddin A, Ambia AR, Elgazzar TA, AlSaud MBM, Kashir J (2021). Application of intravenous immunoglobulin (IVIG) to modulate inflammation in critical COVID-19 - A theoretical perspective. Med Hypotheses.

[65] Milone MC (2005). Treatment of primary Epstein-Barr virus infection in patients with X-linked lymphoproliferative disease using B-cell-directed therapy. Blood.

[66] Fox A (2012). Severe pandemic H1N1 2009 infection is associated with transient NK and T deficiency and aberrant CD8 responses. PLoS One.

[67] Terrell CE, Jordan MB (2013). Perforin deficiency impairs a critical immunoregulatory loop involving murine CD8(+) T cells and dendritic cells. Blood.

[68] Prieto-Perez L (2020). Histiocytic hyperplasia with hemophagocytosis and acute alveolar damage in COVID-19 infection. Mod. Pathol..

[69] Mehta P (2020). COVID-19: consider cytokine storm syndromes and immunosuppression. Lancet.

[70] Papatriantafyllou M (2011). Cytokines: GM-CSF in focus. Nat. Rev. Immunol..

[71] Tan Y (2021). Integrating longitudinal clinical laboratory tests with targeted proteomic and transcriptomic analyses reveal the landscape of host responses in COVID-19. Cell Discov..

[72] Min YQ (2021). Immune evasion of SARS-CoV-2 from interferon antiviral system. Comput Struct. Biotechnol. J..

[73] Hung IF (2020). Triple combination of interferon beta-1b, lopinavir-ritonavir, and ribavirin in the treatment of patients admitted to hospital with COVID-19: an open-label, randomised, phase 2 trial. Lancet.

[74] Bosteels C (2022). Loss of GM-CSF-dependent instruction of alveolar macrophages in COVID-19 provides a rationale for inhaled GM-CSF treatment. Cell Rep. Med.

[75] Bonaventura A (2020). Targeting GM-CSF in COVID-19 Pneumonia: Rationale and Strategies. Front Immunol..

[76] Karakike E, Giamarellos-Bourboulis EJ (2019). Macrophage Activation-Like Syndrome: A Distinct Entity Leading to Early Death in Sepsis. Front Immunol..

[77] Singer M (2016). The Third International Consensus Definitions for Sepsis and Septic Shock (Sepsis-3). JAMA.

[78] Lennartz M, Drake J (2018). Molecular mechanisms of macrophage Toll-like receptor-Fc receptor synergy. F1000Res.

[79] van Egmond M, Vidarsson G, Bakema JE (2015). Cross-talk between pathogen recognizing Toll-like receptors and immunoglobulin Fc receptors in immunity. Immunol. Rev..

[80] Gorelik M, Torok KS, Kietz DA, Hirsch R (2011). Hypocomplementemia associated with macrophage activation syndrome in systemic juvenile idiopathic arthritis and adult onset still’s disease: 3 cases. J. Rheumatol..

[81] Ramanan AV, Schneider R (2003). Macrophage activation syndrome following initiation of etanercept in a child with systemic onset juvenile rheumatoid arthritis. J. Rheumatol..

[82] Sjoelund V, Smelkinson M, Nita-Lazar A (2014). Phosphoproteome profiling of the macrophage response to different toll-like receptor ligands identifies differences in global phosphorylation dynamics. J. Proteome Res.

[83] Bruhns P, Samuelsson A, Pollard JW, Ravetch JV (2003). Colony-stimulating factor-1-dependent macrophages are responsible for IVIG protection in antibody-induced autoimmune disease. Immunity.

[84] Shang L (2014). Selective antibody intervention of Toll-like receptor 4 activation through Fc gamma receptor tethering. J. Biol. Chem..

[85] Delavigne K (2014). Hemophagocytic syndrome in patients with acute myeloid leukemia undergoing intensive chemotherapy. Haematologica.

[86] Teachey DT (2013). Cytokine release syndrome after blinatumomab treatment related to abnormal macrophage activation and ameliorated with cytokine-directed therapy. Blood.

[87] Ishii K (2020). Perforin-deficient CAR T cells recapitulate late-onset inflammatory toxicities observed in patients. J. Clin. Invest.

[88] Xiao X (2021). Mechanisms of cytokine release syndrome and neurotoxicity of CAR T-cell therapy and associated prevention and management strategies. J. Exp. Clin. Cancer Res.

[89] Bertheloot D, Latz E, Franklin BS (2021). Necroptosis, pyroptosis and apoptosis: an intricate game of cell death. Cell Mol. Immunol..

[90] Mucha SR, Rajendram P (2023). Management and Prevention of Cellular-Therapy-Related Toxicity: Early and Late Complications. Curr. Oncol..

[91] Rejeski K (2023). Immune Effector Cell-Associated Hematotoxicity (ICAHT): EHA/EBMT Consensus Grading and Best Practice Recommendations. Blood.

[92] Gust J (2017). Endothelial Activation and Blood-Brain Barrier Disruption in Neurotoxicity after Adoptive Immunotherapy with CD19 CAR-T Cells. Cancer Discov..

[93] Jatiani SS (2020). Myeloma CAR-T CRS Management With IL-1R Antagonist Anakinra. Clin. Lymphoma Myeloma Leuk..

[94] Norelli M (2018). Monocyte-derived IL-1 and IL-6 are differentially required for cytokine-release syndrome and neurotoxicity due to CAR T cells. Nat. Med.

[95] Kelly A, Ramanan AV (2008). A case of macrophage activation syndrome successfully treated with anakinra. Nat. Clin. Pr. Rheumatol..

[96] Lind-Holst M, Hartling UB, Christensen AE (2019). High-dose anakinra as treatment for macrophage activation syndrome caused by refractory Kawasaki disease in an infant. BMJ Case Rep..

[97] Bindoli S, Galozzi P, Doria A, Sfriso P (2023). Intravenous anakinra to curb cytokine storm in adult-onset Still’s disease and in macrophage activation syndrome: A case series. Jt. Bone Spine.

[98] Ajeganova S, De Becker A, Schots R (2020). Efficacy of high-dose anakinra in refractory macrophage activation syndrome in adult-onset Still’s disease: when dosage matters in overcoming secondary therapy resistance. Ther. Adv. Musculoskelet. Dis..

[99] Verbsky JW, White AJ (2004). Effective use of the recombinant interleukin 1 receptor antagonist anakinra in therapy resistant systemic onset juvenile rheumatoid arthritis. J. Rheumatol..

[100] Baker R, Liew JW, Simonson PD, Soma LA, Starkebaum G (2019). Macrophage activation syndrome in a patient with axial spondyloarthritis on adalimumab. Clin. Rheumatol..

[101] Papa R (2020). Successful treatment of refractory hyperferritinemic syndromes with canakinumab: a report of two cases. Pediatr. Rheumatol. Online J..

[102] Olin RL (2008). Successful use of the anti-CD25 antibody daclizumab in an adult patient with hemophagocytic lymphohistiocytosis. Am. J. Hematol..

[103] Lee JH, Ha YJ, Kang EH, Chang SH, Lee YJ (2022). A Case of Macrophage Activation Syndrome During the Treatment of Adult-onset Still’s Disease With Tocilizumab. J. Rheuma. Dis..

[104] Watanabe E (2016). Successful Tocilizumab Therapy for Macrophage Activation Syndrome Associated with Adult-Onset Still’s Disease: A Case-Based Review. Case Rep. Med.

[105] Chellapandian D, Milojevic D (2023). Case report: Emapalumab for active disease control prior to hematopoietic stem cell transplantation in refractory systemic juvenile idiopathic arthritis complicated by macrophage activation syndrome. Front Pediatr..

[106] Gabr JB (2020). Successful treatment of secondary macrophage activation syndrome with emapalumab in a patient with newly diagnosed adult-onset Still’s disease: case report and review of the literature. Ann. Transl. Med.

[107] Prahalad S, Bove KE, Dickens D, Lovell DJ, Grom AA (2001). Etanercept in the treatment of macrophage activation syndrome. J. Rheumatol..

[108] Maeshima K (2012). Adult-onset Still’s disease with macrophage activation syndrome successfully treated with a combination of methotrexate and etanercept. Mod. Rheumatol..

[109] Rivera-Rodriguez L (2021). Use of Infliximab in the Treatment of Macrophage Activation Syndrome Complicating Kawasaki Disease. J. Pediatr. Hematol. Oncol..

[110] Lackner H (2008). Hemophagocytic lymphohistiocytosis as severe adverse event of antineoplastic treatment in children. Haematologica.

[111] Levy O (2022). Ruxolitinib for Refractory Macrophage Activation Syndrome Complicating Adult-Onset Still’s Disease. Eur. J. Rheumatol..

[112] Yi G (2022). Case Report: Baricitinib as an Alternative in the Maintenance Therapy for Macrophage Activation Syndrome Secondary to Nodular Panniculitis. Front Immunol..

[113] Junga Z, Stitt R, Tracy C, Keith M (2017). Novel use of rituximab in macrophage activation syndrome secondary to systemic lupus erythematosus. BMJ Case Rep..

[114] Kishida D (2020). Macrophage activation syndrome in adult dermatomyositis: a case-based review. Rheumatol. Int.

[115] Record JL, Beukelman T, Cron RQ (2011). Combination therapy of abatacept and anakinra in children with refractory systemic juvenile idiopathic arthritis: a retrospective case series. J. Rheumatol..

[116] Eloseily EM (2020). Benefit of Anakinra in Treating Pediatric Secondary Hemophagocytic Lymphohistiocytosis. Arthritis Rheumatol..

[117] Kyriazopoulou E (2017). Macrophage activation-like syndrome: an immunological entity associated with rapid progression to death in sepsis. BMC Med.

